# The microbiome buffers tadpole hosts from heat stress: a hologenomic approach to understand host–microbe interactions under warming

**DOI:** 10.1242/jeb.245191

**Published:** 2023-01-13

**Authors:** Samantha S. Fontaine, Kevin D. Kohl

**Affiliations:** Department of Biological Sciences, University of Pittsburgh, Pittsburgh, PA 15260, USA

**Keywords:** Gut microbes, Thermal tolerance, Transcriptomics, Metagenomics, Amphibians

## Abstract

Phenotypic plasticity is an important strategy that animals employ to respond and adjust to changes in their environment. Plasticity may occur via changes in host gene expression or through functional changes in their microbiomes, which contribute substantially to host physiology. Specifically, the presence and function of host-associated microbes can impact how animals respond to heat stress. We previously demonstrated that ‘depleted’ tadpoles, with artificially disrupted microbiomes, are less tolerant to heat than ‘colonized’ tadpoles, with more natural microbiomes. However, the mechanisms behind these effects are unclear. Here, we compared gene expression profiles of the tadpole gut transcriptome, and tadpole gut microbial metagenome, between colonized and depleted tadpoles under cool or warm conditions. Our goal was to identify differences in host and microbial responses to heat between colonized and depleted tadpoles that might explain their observed differences in heat tolerance. We found that depleted tadpoles exhibited a much stronger degree of host gene expression plasticity in response to heat, while the microbiome of colonized tadpoles was significantly more heat sensitive. These patterns indicate that functional changes in the microbiome in response to heat may allow for a dampened host response, ultimately buffering hosts from the deleterious effects of heat stress. We also identified several specific host and microbial pathways that could be contributing to increased thermal tolerance in colonized tadpoles including amino acid metabolism, vitamin biosynthesis and ROS scavenging pathways. Our results demonstrate that the microbiome influences host plasticity and the response of hosts to environmental stressors.

## Teaser

**Highlighted Article:** Functional changes in the tadpole gut microbiome in response to heat may allow for a dampened host response and ultimately buffer hosts from the deleterious effects of heat stress.

## INTRODUCTION

Increasing global temperatures is one of the largest threats facing animal populations in current times, and the deleterious effects of heat stress have begun to alter organismal physiology (Dillon et al., 2010) and behavior (Beever et al., 2017), ultimately with negative consequences for animal fecundity and survival (Moreno and Møller, 2011). For populations to persist, animals must evolve rapidly to these new conditions or employ phenotypic plasticity if temperatures change too quickly for evolution to occur (Seebacher et al., 2015). Plasticity in organismal gene expression can lead to coordinated and conserved responses that allow animals to better tolerate extreme heat events. For example, heat induces the upregulation of several classes of heat shock proteins (HSPs) which perform a variety of functions to prevent cellular damage from heat, including clearing misfolded proteins, repairing DNA damage, and inducing transcription of other stress response pathways (Richter et al., 2010). These responses ultimately act to increase animal survivorship under thermal stress (Harada and Burton, 2019). Gene expression responses to heat can vary from relatively few genes to large transcriptome-wide shifts in expression (Logan and Cox, 2020). However, we still lack a complete understanding of how changes in gene expression in response to heat translate to changes in whole-animal performance or fitness. Specifically, there is not always concordance between the magnitude of gene expression plasticity in response to heat and the shift in whole-organism heat tolerance (Logan and Cox, 2020). Therefore, there may be other factors that play a role in regulating animal responses to heat.

In addition to host responses to the environment, animals harbor dense and diverse communities of microorganisms (bacteria, fungi, archaea, etc.), collectively termed the microbiome, that influence their host's physiology, resulting in ‘emergent phenotypes’ (Lynch and Hsiao, 2019) that can enhance phenotypic plasticity and shape how organisms respond to environmental challenges (Alberdi et al., 2016). For example, in mammalian herbivores, gut microbial communities help hosts break down difficult-to-digest plant material and facilitate feeding on toxic food sources through the breakdown of plant secondary compounds (Kohl et al., 2014). Gut microbial communities of several invasive amphibian species have been shown to be exceptionally plastic in response to environmental change and may help facilitate host adaptation to novel environments (Wagener et al., 2020 preprint; Fontaine and Kohl, 2020; Zhou et al., 2020 preprint). Additionally, in hibernating mammals, exposure to cold can change the gut microbiota, and these changes result in increased energy acquisition, cold tolerance of the host, and nutritional balance during hibernation (Sommer et al., 2016; Chevalier et al., 2015; Regan et al., 2022). By altering host phenotypes in these ways, the microbiome can ultimately shape host responses to selective pressures, and the evolutionary trajectories of populations (Henry et al., 2021).

In ectothermic animals, host colonization with individual microbial symbionts as well as more complex microbial communities can have protective effects when animals face heat stress (Hector et al., 2022). In invertebrate systems, individual microbial symbionts can increase host growth rates (Harmon et al., 2009; Russell and Moran, 2006), fecundity (Hoang et al., 2019; Montllor et al., 2002) and survival under heat stress conditions (Russell and Moran, 2006; Nakagawa et al., 2016; Brumin et al., 2011; Berkelmans and Van Oppen, 2006), as well as their behavioral preference for warm environments (Porras et al., 2020) and the upper lethal limits of thermal tolerance (critical thermal maximum, CT_max_) (Porras et al., 2020). These effects can ultimately facilitate host adaptation to thermally stressful environments (Hoang et al., 2021). In invertebrates with more complex microbial communities, gut microbial transplants from flies reared at cooler temperatures reduce the CT_max_ of recipient flies (Moghadam et al., 2018), and axenic flies exhibit poorer survival than conventionalized flies under heat stress (Jaramillo and Castañeda, 2021). Mechanistically, the protective effects of microbes for invertebrate hosts under heat stress involve the colonization-mediated stimulation of host gene upregulation of oxidative stress response pathways (Nakagawa et al., 2016), cytoskeleton genes that protect cellular integrity (Brumin et al., 2011), and HSPs (Porras et al., 2020). Additionally, bacterially derived metabolites and HSPs may also act to increase their host's heat tolerance directly (Burke et al., 2010; Dunbar et al., 2007).

In a vertebrate system, we previously demonstrated a link between the gut microbiota of an ectothermic vertebrate and the host's heat tolerance (Fontaine et al., 2022). Specifically, we raised tadpoles of the green frog (*Lithobates clamitans*) in two conditions which differed in their environmental microbial exposure. ‘Colonized’ tadpoles were raised in natural pond water and had gut microbiomes that were significantly more diverse and differed in composition from those of ‘depleted’ tadpoles, which were raised in autoclaved pond water to reduce the microbial diversity of this environmental source for inoculation (Fontaine et al., 2022). Compared with colonized tadpoles, depleted tadpoles had lower acute heat tolerance measured via CT_max_, poorer locomotor performance at high temperatures, and ultimately lower survival under heat stress conditions (32–34°C) (Fontaine et al., 2022), indicating that commensal microbial communities can also influence host heat tolerance in vertebrate hosts. These results were consistent with previous correlative studies in lizards showing associations between gut microbiota composition and host CT_max_ (Moeller et al., 2020), and microbiota diversity with animal survival under warming conditions (Bestion et al., 2017). Interestingly, depleted tadpoles also had lower activity of aerobic mitochondrial enzymes and reduced metabolic rates at high temperatures in large-bodied individuals (Fontaine et al., 2022), which could explain differences in their thermal tolerance (Pörtner et al., 2017). However, the mechanisms governing the relationship between ectothermic vertebrate gut microbial communities and host heat tolerance are far less resolved than those among invertebrate hosts.

Here, we use a ‘hologenomics’ approach to identify potential mechanistic pathways that may underlie the relationships observed between tadpole heat tolerance and their gut microbial communities. Specifically, we analyzed the host gut transcriptome and tadpole gut microbial metagenome in colonized and depleted *L. clamitans* tadpoles under non-stressful or heat stress conditions. Such hologenomic approaches have been called for to answer outstanding questions regarding the evolution of relationships between hosts and microbes (Alberdi et al., 2022), and have been used previously to understand the influence of microbes on various host phenotypes including blood feeding (Mendoza et al., 2018) and the degradation of complex plant material (Bredon et al., 2018). Here, we expand on this approach by assaying host gene expression and microbial metagenomics under variable conditions with the major goal of identifying host and microbial pathways that differ in their responses to heat between colonized and depleted tadpoles, as these differences may underlie the previously observed reductions in heat tolerance of depleted tadpoles. Additionally, we identify host and microbial pathways that differ in response to temperature alone (regardless of colonization effects) and microbial colonization treatment alone (regardless of temperature effects).

## MATERIALS AND METHODS

### Frog spawning and tadpole development

The tadpoles used in these experiments were generated in parallel with animals used for previously published work, where detailed rearing and development methods can be found (Fontaine et al., 2022). In brief, mating pairs of adult green frogs, *Lithobates clamitans* (Latreille 1801), were collected from a pond at Pymatuning Laboratory of Ecology (Linesville, PA, USA) in July 2020 and were transported to the laboratory at University of Pittsburgh. Permission to collect animals was obtained from Pennsylvania Fish and Boat Commission under scientific collector's permit 2020-01-0131. Animal research was approved by the University of Pittsburgh IACUC (protocol #18062782). Additionally, 100 l of pond water was collected monthly from the same pond for the duration of the experiment and stored in the laboratory at 4°C after filtering through a 500 µm sieve. We induced spawning with a hormonal injection in a single pair of adult frogs using previously described methods (Fontaine et al., 2022; Trudeau et al., 2010). The resulting embryos were maintained in autoclaved laboratory water until they developed to Gosner stage 25 (Gosner, 1960). Tadpoles were then distributed evenly into two groups with differing environmental microbial community exposure. Colonized tadpoles were maintained individually in 12 oz (∼0.35 l) polypropylene containers filled with 75 ml (25% volume) unmanipulated pond water from their parent's site of capture and 225 ml (75% volume) autoclaved laboratory water. Depleted tadpoles were maintained in identical conditions, except that the pond water they were exposed to was also autoclaved. The environmental chamber was set to 22°C, 65% humidity, and a 14 h:10 h light:dark cycle. Tadpoles were transferred to fresh water of the appropriate treatment weekly and fed a diet of autoclaved rabbit chow suspended in autoclaved agar and supplemented with a commercial pet vitamin *ad libitum*. Tadpoles developed in these conditions for 9 weeks.

### Heat stress and sample collection

After tadpole development, 10 colonized and 10 depleted tadpoles were transferred to a second environmental chamber set to 32°C with the same humidity and light cycle, while all other tadpoles remained at 22°C. Tadpoles were exposed to this warmer temperature for a period of 24 h. After this period, 10 colonized and 10 depleted tadpoles from both the 32°C and 22°C treatments were removed from the experiment and euthanized by double pithing. Chemical euthanasia agents (e.g. MS-222) were not used in this particular experiment because they can alter the expression of stress response genes, including HSPs (Yu et al., 2018). We recorded each tadpole's body mass (g), body length (mm) and Gosner stage. Subsequently, each animal was dissected to remove the entire gastrointestinal tract. We chose to use the gut for gene expression and metagenomic analyses because it is the site of highest microbial density in vertebrates (Sender et al., 2016), the gut microbiota are important for tadpole physiological function (Pryor and Bjorndal, 2005), and heat can induce changes in gene expression in the gut of amphibians (Near et al., 1990) and other vertebrates (He et al., 2018; Pearce et al., 2015). We unraveled each coiled gut and split it into two samples, with effort to equally distribute longitudinal sections of the gut across tubes. Samples used for transcriptomics were placed in RNAlater for 24 h at 4°C, and then frozen until processing at −80°C after the removal of RNAlater. Samples used for metagenomics were flash frozen with liquid nitrogen and then frozen at −80°C until processing. All dissection instruments were flame sterilized between individuals.

### Host transcriptomics: sample preparation and sequencing

From the samples collected for transcriptomics above, we chose five samples from each of the four treatment groups to be used for host RNA sequencing. Our previous work demonstrated significant differences in body size and development between colonized and depleted tadpoles, though observed differences in temperature tolerance were independent of these effects (Fontaine et al., 2022). Regardless, to avoid potential confounding effects of size or development on gene expression (Row et al., 2016), we selected individuals for this experiment that were of similar size and developmental stage (Table S1). From these individuals, we extracted RNA from gut samples using the RNeasy Plus Mini Kit (Qiagen, Hilden, Germany) following the manufacturer's protocol. At step 3 of the protocol, we used 350 µl of buffer RLT and a TissueLyser II (Qiagen) set to 30 Hz for 1 min for disruption and homogenization. We quantified the concentration of RNA (ng µl^−1^) and the 260 nm/280 nm absorbance ratio in each sample using a plate reader and stored samples at −20°C until sequencing. Extracted RNA samples were sent to the University of Pittsburgh Genomics Research Core (Pittsburgh, PA, USA) for library preparation. Briefly, mRNA was isolated, cDNA libraries constructed, and unique sample indexes added using the TruSeq RNA Library Prep Kit (Illumina, San Diego, CA, USA) following the manufacturer's protocol. Libraries were pooled within samples and sent to the University of Pittsburgh Medical Center Genome Center (Pittsburgh, PA, USA) for sequencing on the Illumina NovaSeq to generate 2×101 bp paired-end reads. Raw RNA-seq reads were trimmed to remove indexes and for quality using Trim Galore (https://www.bioinformatics.babraham.ac.uk/projects/trim_galore/). Reads were mapped using BWA (Li and Durbin, 2009) to an available reference draft genome of the green frog's congener, the American bullfrog (*Lithobates catesbeiana*, GenBank accession GCA_002284835.2) (Hammond et al., 2017). We additionally mapped our reads to a reference genome of the African clawed frog (*Xenopus laevis*, GenBank accession GCA_017654675.1); however, using the *X. laevis* genome, <60% of reads per sample were successfully aligned. Using the bullfrog genome, >97% of reads were successfully aligned to the reference genome for each sample and, thus, this alignment was used for all downstream analyses. Next, we used StringTie (Pertea et al., 2015) to generate a matrix of read counts across samples for each transcript. We removed any transcripts from analysis that were present at an abundance of <2 counts per million or in fewer than three samples. This step reduced the final dataset from 410,385,578 reads over 22,238 transcripts to 392,568,234 reads over 8477 transcripts.

### Microbiome metagenomics: sample preparation and sequencing

To prepare samples for metagenomics, we separated the gut contents from the gut tissue for each sample and extracted DNA from the samples using the QIAmp PowerFecal Pro DNA Kit (Qiagen) following the manufacturer's protocol. We quantified the concentration of DNA (ng µl^−1^) and the 260 nm/280 nm absorbance ratio in each sample using a plate reader and stored samples at −20°C until sequencing. If gut samples did not contain a sufficient amount of gut contents for extraction (some guts were nearly empty), the samples were not used. Of the samples we successfully extracted, we selected the five from each of the four treatment groups with the highest DNA concentration to be used for metagenomic analysis. Some of these samples were from the same individuals from which we obtained transcriptomic samples (Table S1); however, we obtained too few paired samples to integrate these data in downstream analyses. The selected samples were sent to Diversigen (New Brighton, MN, USA) for shallow shotgun metagenomic sequencing using their BoosterShot method. Briefly, libraries were prepared using the Nextera XT DNA Library Preparation Kit (Illumina) and sequenced on the Illumina NovaSeq generating 2×100 bp paired-end reads. Reads were trimmed and filtered for low quality or length, and using BURST (Al-Ghalith and Knights, 2020 preprint), were aligned to Diversigen's Venti database, which contains RefSeq bacterial genomes and an annotated bacterial KEGG database (Kanehisa and Goto, 2000), at 97% similarity. Count tables of KEGG orthologs were then created for each sample.

### Statistical analyses

#### Host transcriptomics

To compare differences in overall gene expression profiles across the four treatment groups, we created a Bray Curtis distance matrix in the R Vegan package (Dixon, 2003) from our filtered count data of all expressed transcripts (8477 total), and visualized these data using principal coordinates analysis (PCoA). We assessed the statistical significance of the effect of microbial colonization, temperature or their interaction on tadpole gut gene expression with a PERMANOVA using the adonis2 function and 999 permutations. Next, we identified genes that were differentially expressed based on temperature only (warm versus cool across all tadpoles), on colonization only (colonized versus depleted across all tadpoles) or on temperature within colonized or depleted tadpoles separately (warm versus cool within each colonization group) using the R package edgeR (Robinson et al., 2010). *P*-values were corrected using the Benjamini–Hochberg false discovery rate (BH FDR). To functionally annotate all differentially expressed genes, for each transcript identified as differentially expressed in any of the comparisons described above, we used Blast2GO (version 6.0.3) (Conesa et al., 2005) to BLAST the sequence against the NCBI database and identify the associated GO functions using the SWISSPROT database with the blastx-fast function (Blast E-value=0.001).

Next, we used enrichment analyses to identify gene ontology (GO) terms that were enriched among differentially expressed genes across our comparisons of interest. Because there were very few differentially expressed genes between colonized and depleted tadpoles (without considering temperature), we did not perform an enrichment analysis for this comparison. We created lists of genes that were upregulated or downregulated in response to heat in all tadpoles, colonized tadpoles only, and depleted tadpoles only. We then used Fisher's exact tests with BH FDR corrected *P*-values in Blast2GO to identify GO terms that were differentially enriched across groups. To identify pathways enriched in response to heat across all tadpoles, we compared gene lists (upregulated or downregulated in response to heat) with all genes in the dataset. To identify pathways differentially enriched between colonized or depleted tadpoles in response to heat, we compared upregulated and downregulated gene lists of colonized and depleted tadpoles with one another. For any significant comparisons, we used the ‘Reduce to Most Specific’ function within Blast2GO, which removes more general terms and retains the most specific statistically significant GO terms.

#### Microbiome metagenomics

We removed one depleted sample from the warm temperature group from all analyses because of exceptionally low numbers of KEGG orthologs (KOs) identified in the sample (number of KOs=33 and mean±s.e.m. of KOs for all other samples=10,801±3709). To identify overall differences in microbial community function across groups, we created a Bray–Curtis distance matrix based on KO counts for each sample in R in the vegan package and used a PERMANOVA to statistically compare the effects of temperature, microbial colonization treatment, and their interaction on microbial function using the adonis2 function with 999 permutations. We visualized these data using a PCoA. Next, KEGG orthologs were collapsed into KEGG modules, and we used the program STAMP (Parks et al., 2014) to identify differentially abundant modules across temperature groups, colonization groups, and temperature groups within colonization groups (warm versus cool in colonized or depleted tadpoles separately). Within STAMP, we used the two-group analysis option and compared KEGG module abundance between groups using a Welch's *t*-test, correcting *P*-values with the BH FDR method. For all comparisons, we chose the option to use unclassified reads only when calculating frequency profiles.

## RESULTS

**Fig. 1.**
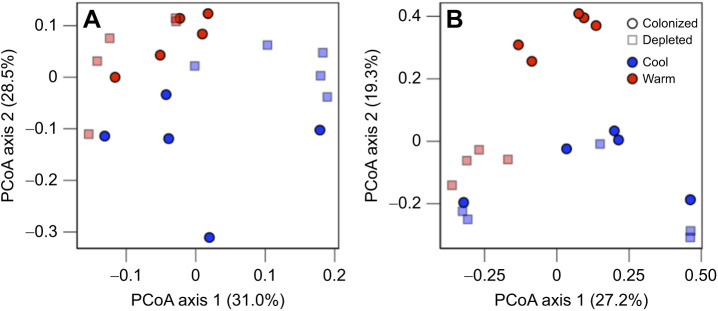
**Comparison of tadpole gut transcriptome and gut microbial metagenome responses to heat.** Principal coordinate analysis (PCoA) plots created using Bray–Curtis distance matrices to compare (A) the host gut transcriptome of all expressed transcripts and (B) the gut microbial metagenome of all functional KEGG orthologs across colonized and depleted tadpoles in cool and warm treatment groups. Within graphs, colonization treatment is denoted by symbol, while temperature treatment is denoted by color. Percentages represent the proportion of variation explained by each axis. *N*=5 individuals per group, except for the warm depleted group in B, where *N*=4. The host gut transcriptome was significantly impacted by temperature, and the interaction between temperature and microbial colonization treatment (PERMANOVA, *P*<0.01 for both). The gut microbial metagenome was significantly impacted by temperature, microbial colonization treatment and the interaction of these variables (PERMANOVA, *P*<0.05 for all).

Overall, we observed differences in the gut transcriptome and gut microbial metagenome of tadpoles based on both temperature and microbial colonization treatment (Fig. 1). Specifically, temperature and the interaction between temperature and microbial colonization treatment impacted the host transcriptome (Fig. 1A; PERMANOVA, temperature *F*=5.37, temperature×colonization *F*=3.30, *P*<0.01 for both). Temperature, microbial colonization and their interaction impacted the microbial metagenome (Fig. 1B; PERMANOVA, temperature *F*=3.70, colonization *F*=2.61, temperature×colonization *F*=2.26, *P*<0.05 for all). We explore the more detailed analyses that were performed for the host transcriptome and microbial metagenome separately below.

### Host transcriptome

Considering the effects of temperature alone, we identified 1733 genes that were significantly upregulated or downregulated in response to heat stress across all tadpoles (Table 1; full gene lists and statistics are given in Table S2). Within genes upregulated in response to heat, there were five GO terms that were significantly enriched compared with the rest of the dataset (Table S2). These included rRNA processing, RNA modification, tRNA processing, negative regulation of mRNA splicing and mitochondrial translation. Within genes downregulated in response to heat, there were 108 GO terms that were significantly enriched compared with the rest of the dataset (Table S2). The five most highly significant of these terms were positive regulation of transcription by RNA polymerase II, negative regulation of transcription by RNA polymerase II, positive regulation of NF-κB transcription factor activity, protein ubiquitination and positive regulation of protein catabolic processes.

**Table 1.**
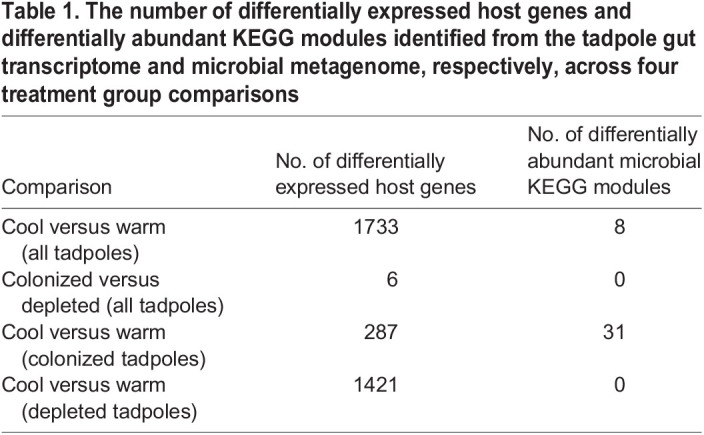
The number of differentially expressed host genes and differentially abundant KEGG modules identified from the tadpole gut transcriptome and microbial metagenome, respectively, across four treatment group comparisons

In response to microbial colonization alone, we identified only six genes (three upregulated and three downregulated) that were differentially expressed in depleted tadpoles compared with colonized tadpoles (Table 1; full gene lists and statistics are given in Table S2). Notably, the three downregulated genes were cytochrome P450 genes associated with lipid hydroxylation and/or xenobiotic metabolism.

**Fig. 2.**
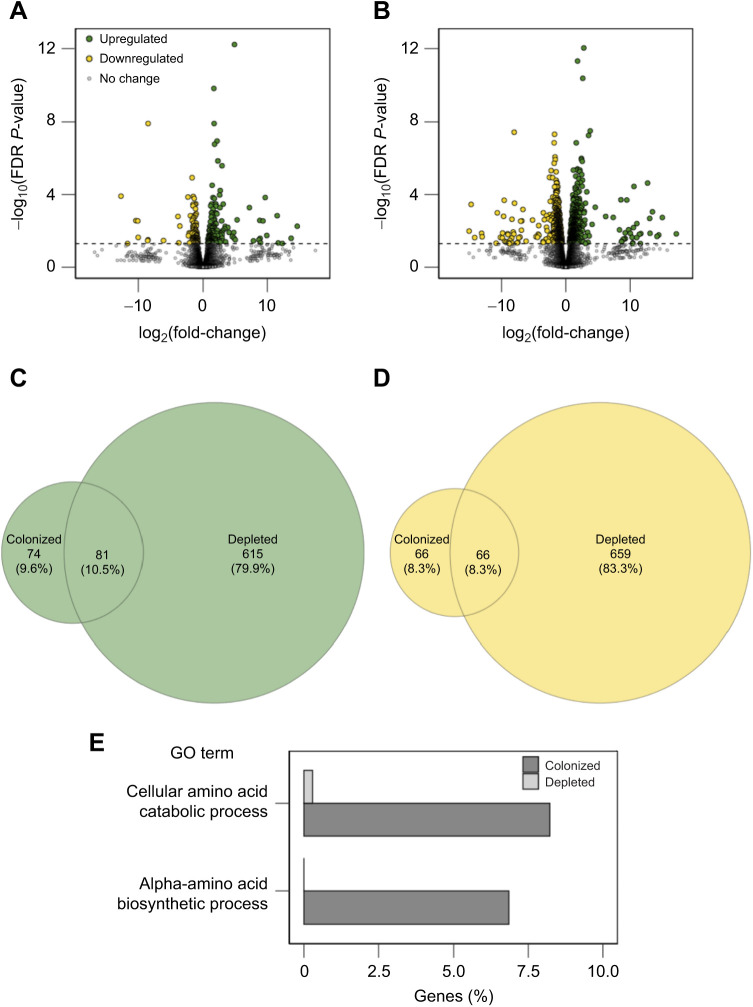
**Comparison of colonized and depleted tadpole gut transcriptome responses to heat.** (A,B) Volcano plots for (A) colonized and (B) depleted tadpoles showing genes upregulated (green), downregulated (yellow) or not affected (gray) in response to heat. The *y*-axes demonstrate the significance (*P*-value) thresholds at the dotted lines and the *x*-axes show the direction and magnitude of each gene's change in expression. (C,D) For all genes that were either (C) upregulated or (D) downregulated in response to heat, Venn diagrams show the number of genes that were differentially expressed in colonized tadpoles only, depleted tadpoles only, or in both groups. The areas of each section in the Venn diagrams are weighted by the number of genes in those sections. (E) Gene ontology (GO) terms, and the percentage of genes identified under those terms, that were significantly differentially enriched (false discovery rate, FDR *P*<0.05) among genes upregulated in colonized tadpoles compared with those upregulated in depleted tadpoles upon exposure to heat based on Fisher's exact tests. *N*=5 individuals per group.

When considering colonized and depleted tadpoles separately, depleted tadpoles differentially expressed more host genes in response to heat (1421 genes total) than colonized tadpoles did (287 genes total), and this trend was true for both upregulated and downregulated genes (Table 1, Fig. 2A,B; full gene lists and statistics are given in Table S2). We created Venn diagrams to depict the number genes upregulated and downregulated in response to heat that were shared between colonized and depleted tadpoles. We found that only 10.5% and 8.3% of upregulated and downregulated genes, respectively, were shared between the two colonization groups (Fig. 2C,D). The majority of upregulated genes (79.9%) and downregulated genes (83.3%) were unique to depleted tadpoles; however, we still observed several upregulated genes (9.6%) and downregulated genes (8.3%) that were unique to colonized tadpoles (Fig. 2C,D), indicating substantial differences in host responses to heat depending on microbial colonization treatment. In downregulated genes, there were no differences in functional enrichment between colonized and depleted tadpoles in response to heat. However, we identified two GO terms that were significantly enriched in upregulated genes in colonized tadpoles as compared with depleted tadpoles exposed to heat (Fig. 2E; Fisher's exact test, FDR *P*<0.05 for both). Specifically, genes related to cellular amino acid catabolic processes and alpha-amino acid biosynthetic processes accounted for 8.2% and 6.8% of upregulated genes, respectively, in colonized tadpoles in response to heat. Of the genes that were assigned to these categories within colonized tadpoles, 57% were present in both categories. In depleted tadpoles, only 0.29% of genes upregulated in response to heat were related to cellular amino acid catabolic processes, and none were related to alpha-amino acid biosynthetic processes (Fig. 2E).

### Microbiome metagenome

In response to temperature alone, we identified eight microbial KEGG modules that were differentially abundant between cool and warm temperatures (Table 1; full module list and statistics are given in Table S3). Of these eight KEGG modules, six were found within the higher order KEGG pathway of metabolism, and four of these were specifically related to carbohydrate metabolism and tended to be present in greater abundance in the cool temperature group. When considering microbial colonization treatment alone, we did not identify any KEGG modules that were differentially abundant between colonized and depleted tadpoles (Table 1).

**Fig. 3.**
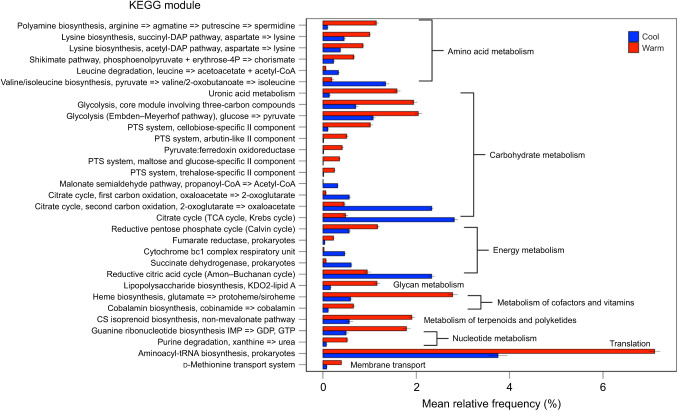
**KEGG modules in the colonized tadpole gut microbial metagenome identified as differentially abundant in response to heat.** Differential abundance (FDR *P*<0.05) of KEGG modules between cool (blue) and warm (red) treatment groups based on Welch's *t*-tests. Modules are grouped by their higher order KEGG pathways, and subsequently by the temperature at which they were most abundant. Bars and error bars represent the mean±s.e.m. relative frequency of each module. *N*=5 individuals per group.

When comparing colonized and depleted tadpole microbiome metagenomes separately, we observed that the colonized tadpole microbiome was more functionally responsive to heat than the depleted tadpole microbiome. Specifically, 31 KEGG modules were differentially abundant between cool and warm groups in the colonized tadpole metagenome (Table 1, Fig. 3; full module list and statistics are given in Table S3), while no KEGG modules were differentially abundant between temperature groups in depleted tadpoles. Of the heat-sensitive pathways in colonized tadpoles, 29 of 31 were classified within the higher order KEGG pathway of metabolism which included amino acid metabolism, carbohydrate metabolism, energy metabolism, glycan metabolism, metabolism of cofactors and vitamins, metabolism of terpenoids and polyketides, and nucleotide metabolism. The most differentially abundant modules were found within carbohydrate metabolism.

## DISCUSSION

The main goal of our study was to identify differences in host and microbial responses to heat stress between colonized (more diverse, natural microbiome) and depleted (less diverse, artificially disrupted microbiome) tadpoles which may underlie the previous observation that depleted tadpoles are less tolerant to heat than colonized tadpoles (Fontaine et al., 2022). We additionally wanted to understand the impacts of both heat and microbial colonization treatments on tadpole host and microbial function independently of one another.

The largest number of differentially expressed host genes we observed across any comparison was between cool and warm temperature groups among all tadpoles (Table 1), consistent with the consensus that heat induces large physiological changes in ectothermic animals (Huey and Kingsolver, 1989). Similar to studies in other ectothermic animal groups, we found that heat-sensitive genes were enriched for functions related to transcription and translation, as well as protein processing (Logan and Cox, 2020) (Table S2). Interestingly, however, we did not observe differential expression in any host HSP genes in response to temperature, which is extremely common across ectotherms in general (Logan and Cox, 2020) and among amphibians in particular (Ali et al., 1997). We also did not observe any changes in microbial HSPs upon exposure to heat, which are known to influence host heat tolerance (Burke et al., 2010; Dunbar et al., 2007), but did observe several changes to microbial metabolism, and specifically carbohydrate metabolism (Table S2), under heat stress, which is consistent with other studies predicting the effects of heat on microbiome function (Fontaine and Kohl, 2020; Ziegler et al., 2017).

We observed few host genes whose expression was impacted by microbial colonization treatment alone (Table 1). Studies in lab mice show extensive differences in host gene expression between germ-free and conventionalized states; however, gene expression profiles are relatively similar across mice colonized with microbiota from different sources (mice versus zebrafish) (Rawls et al., 2006). In our system, all tadpoles are colonized with a microbiome, albeit microbiomes of different composition, and thus, if microbial colonization alone, and not microbial identity, impacts host gene expression, it is not surprising that we observed few differences across this comparison. A trend we did observe was a consistent downregulation of cytochrome P450 genes in depleted tadpoles compared with colonized tadpoles (Table S2). Each of these genes were within the cytochrome P450 CYP2 enzyme family, which are important in xenobiotic detoxification and metabolism (Kubota et al., 2011). Tadpoles readily ingest high concentrations of toxic plant secondary compounds through their herbivorous foraging strategy (Radanovic et al., 2017), and the cytochrome P450 CYP2 family is important for metabolizing toxins from herbivorous diets in other animals (Greenhalgh et al., 2021). These enzymes also play an important role in lipid hydroxylation and aid in the breakdown of dietary fatty acids (Oliw, 1994), and interestingly, we observed differences between the lipid profiles of colonized and depleted tadpoles previously (Fontaine et al., 2022). It would be interesting to manipulate the diets of colonized and depleted tadpoles with varying levels of dietary toxins and/or lipids to understand the functional consequences of these gene expression changes.

When considering the joint impacts of temperature and microbial colonization treatment, one major trend we observed was a difference in the degree of both host and microbiome plasticity in response to heat stress between tadpole microbial colonization treatment groups. In response to heat, depleted tadpoles exhibited more plastic host gene expression than colonized tadpoles. Specifically, in their gut, depleted tadpoles differentially expressed ∼5 times more genes than colonized tadpoles in response to heat (Table 1, Fig. 2A,B), and very few of these genes were shared with those upregulated or downregulated by colonized tadpoles (Fig. 2C,D). Although phenotypic plasticity in response to environmental change is often assumed to be beneficial, large numbers of genes that are actually maladaptive for heat tolerance can be differentially expressed in response to heat in ectotherms (Campbell-Staton et al., 2021). In fact, it is possible that the greater host transcriptomic response we observed in depleted tadpoles is indicative of greater stress caused by the heat treatment, as more dampened gene expression responses are often observed in environmental stress-tolerant populations as compared with environmental stress-sensitive populations (Rivera et al., 2021).

In contrast to host gene expression responses, we observed greater plasticity in microbiome function in response to heat in colonized tadpoles compared with depleted tadpoles. Specifically, we observed 31 functional KEGG modules that differed in abundance across temperature treatments in colonized tadpole microbiomes (Table 1, Fig. 3), while there were zero KEGG modules that were differentially abundant between temperature treatments in depleted tadpole microbiomes. These results are congruent with the idea that the microbiome can buffer hosts from environmental stress, such that beneficial changes in microbiome function enable dampened host responses to stressful conditions, and ultimately lead to better outcomes for host fitness than relying on host responses alone (Timm et al., 2018).

Despite responding differently to heat, we did not observe any differences in microbial functional profiles between colonized and depleted tadpoles when analyzing the effects of microbial colonization alone (Table 1). We previously showed that depleted tadpoles do still host microbes; however, their gut microbiomes are less diverse and differ in composition compared with those of colonized tadpoles based on amplicon sequencing of the bacterial 16S rRNA gene (Fontaine et al., 2022). Functional redundancy in host-associated microbial communities is common and microbial communities that are disparate taxonomically may appear similar functionally (Moya and Ferrer, 2016). However, taxonomically different microbes with the same metabolic potential may still respond differently (e.g. enzyme efficiency, growth rate) when faced with changing environmental conditions (Louca et al., 2018). Thus, we hypothesize that microbes in depleted tadpole microbiomes may be more heat sensitive in terms of their growth or survival than those of colonized tadpoles, explaining why they could appear functionally similar to colonized microbiomes but be unable to respond to heat in the same way.

In addition to quantifying plasticity in terms of the number of genes or bacterial KEGG modules affected by heat, we sought to understand some functional implications of these changes. We identified two GO term pathways related to amino acid catabolism and anabolism (Fig. 2E) that were enriched in the host transcriptomic response to heat within colonized tadpoles compared with depleted tadpoles. Genes related to protein synthesis are commonly upregulated among ectotherms in response to heat (Logan and Cox, 2020), and free amino acids may be important energy sources under warming (Tripp-Valdez et al., 2017). Gut microbes have been shown to be important to their host's overall amino acid metabolism (Matsumoto et al., 2012; Hooper et al., 2002), and hosts often utilize bacterially derived amino acids (Metges, 2000). For example, up to 20% of host lysine can be derived from gut microbial sources (Metges, 2000). Of the 31 microbial functions that differed between warm and cool conditions in colonized tadpoles, six were related to amino acid metabolism, and two of these showed increases in microbial lysine biosynthetic pathways under warm conditions (Fig. 3). It is possible that crosstalk between hosts and the gut microbiome related to amino acid metabolism facilitated increased host gene expression of amino acid anabolic and catabolic pathways in colonized tadpoles, ultimately increasing the host's tolerance to increased temperature. In contrast, depleted tadpoles may lack the necessary microbes to facilitate this process, ultimately resulting in their lowered heat tolerance.

Additionally, the gut microbiome of colonized tadpoles exhibited some specific responses to heat that could impact the thermal tolerance of tadpole hosts. For example, in the colonized tadpole microbiome, functions involved in the biosynthesis of cobalamin (vitamin B_12_) were more abundant under warm conditions (Fig. 3). In the algal species *Chlamydomonas reinhardtii*, cobalamin-producing bacteria are important in maintaining the host's thermal tolerance under warming because of the need for cobalamin for host methionine production (Xie et al., 2013). Methionine is an amino acid which modulates algal thermal tolerance through protein synthesis and maintenance of growth rate under high temperatures, and activation of heat shock responses (Xie et al., 2013). Interestingly, we also observed an increase in functions related to the bacterial d-methionine transport system in the colonized tadpole microbiome in warm conditions (Fig. 3), and expression of these bacterial transport proteins has been previously related to the level of methionine present (Gál et al., 2002).

We also observed increases in microbial sequences related to the enzyme pyruvate:ferredoxin oxidoreductase in colonized tadpoles under warm conditions (Fig. 3). This enzyme reduces ferredoxin, an important mediator of reactive oxygen species (ROS) scavenging pathways, overexpression of which ultimately increases survival under heat stress in *C. reinhardtii* (Lin et al., 2013). Interestingly, colonized tadpoles show greater activity of mitochondrial enzymes at high temperatures compared with depleted tadpoles (Fontaine et al., 2022), and this activity could increase production of ROS and the need for ROS scavengers (Freeman and Crapo, 1981). Increased abundance of these example bacterial functions under warm conditions may contribute to the greater heat tolerance of colonized tadpoles compared with depleted tadpoles, which lack the enrichment of these pathways under heat stress. However, it is currently unclear whether similar pathways govern heat tolerance in our study tadpoles to those in organisms such as algae. To test some of these described hypotheses, future experiments could focus on manipulating suspected pathways (e.g. dietary supplemented amino acids or vitamins) and observing how the treatments impact host heat tolerance.

In summary, depleted tadpoles, which are less heat tolerant, exhibited high plasticity in host transcriptome responses to heat, while colonized tadpoles, which are more heat tolerant, exhibited greater microbiome plasticity in response to heat. The interactions of several host and microbial pathways may explain these effects, including amino acid metabolism, vitamin biosynthesis and ROS scavenging pathways. Our findings demonstrate that the composition of a host's microbial community can impact the capacity for host phenotypic plasticity. Importantly, this plasticity may not always be beneficial for the host and can actually be maladaptive as evidenced by high levels of plasticity observed in depleted tadpoles, along with reduced heat tolerance. In contrast, we observed plasticity in the microbiome to be beneficial to host outcomes as evidenced by greater degrees of microbiome plasticity in colonized tadpoles, which exhibit increased heat tolerance. Overall, these results suggest hosting a microbial community that is functionally responsive to heat can help buffer hosts from deleterious effects of heat stress. Thus, rather than strictly a host response to environmental conditions, heat tolerance, and perhaps plasticity more generally, may represent an emergent phenotype that is governed in some way by interactions between hosts and their microbes (Lynch and Hsiao, 2019). As global temperatures continue to rise, it will be important to incorporate host–microbe interactions into our understanding of host responses to climate change, as these interactions may ultimately alter evolutionary responses to warming conditions (Henry et al., 2021).

## Supplementary Material

10.1242/jexbio.245191_sup1 Supplementary information
