# Supplementary material for: The microbiome buffers tadpole hosts from heat stress: a hologenomic approach to understand host–microbe interactions under warming
Source: J Exp Biol. 2023 Jan 13;226(1):jeb245191. doi: 10.1242/jeb.245191 (PMC10086385; doi:10.1242/jeb.245191)
Supplement: Supplementary information [file jexbio-226-245191-s1.pdf]

Table S1. Sample identifiers, treatment group categories, and tadpole morphometrics for all samples used for transcriptomics and metagenomics analyses. Sample IDs in bolded red text came from individual tadpoles that were used in both analyses.

Click here to download Table S1

Table S2. ferentially expressed host genes, their associated GO terms, and associated statistics for comparisons between several treatment group comparisons (sheets 1-4), and lists of significantly enriched GO terms and associated statistics for two treatment group comparisons (sheets 5-6).

Click here to download Table S2

Table S3. Lists of significantly differentially abundant KEGG modules in the microbial metagenome, and associated statistics for comparisons between several treatment group comparisons (sheets 1-2).

Click here to download Table S3

Journal of Experimental Biology • Supplementary information
